# Residual TPO Content of Photopolymerized Additively Manufactured Dental Occlusal Splint Materials

**DOI:** 10.3390/biomedicines13010044

**Published:** 2024-12-27

**Authors:** Philipp Messer-Hannemann, Max Wienhold, Hoda Esbak, Alexander Brunner, Andreas Schönebaum, Falk Schwendicke, Susanne Effenberger

**Affiliations:** 1 DMG Dental-Material Gesellschaft mbH, 22547 Hamburg, Germany; mwienhold@mtcompanies.com (M.W.); hesbak@mtcompanies.com (H.E.); abrunner@mtcompanies.com (A.B.); aschoenebaum@mtcompanies.com (A.S.); 2 Department of Conservative Dentistry and Periodontology, LMU Klinikum, 80336 Munich, Germany; falk.schwendicke@med.uni-muenchen.de (F.S.); susanne.effenberger@med.uni-muenchen.de (S.E.)

**Keywords:** 3D printing, additive manufacturing, occlusal splints, photoinitiator, TPO, extraction

## Abstract

**Background/Objectives**: Diphenyl (2,4,6-trimethylbenzoyl) phosphine oxide (TPO) is widely used in the dental industry as a photoinitiator for resin-based materials, while its use may be further limited given its toxicological risks. The aim of this study was, therefore, to analyze the residual TPO content of 3D-printed resin-based dental splint materials. **Methods**: Six resin-based splint materials were analyzed: LuxaPrint Ortho Plus (DMG), FREEPRINT splint 2.0 (Detax), optiprint splint (Dentona), KeySplint Soft (KeyPrint), FREEPRINT ortho (Detax), V-Print splint comfort (Voco). Grid-shaped specimens were fabricated using the recommended workflow of each manufacturer (n = 18). TPO extraction was conducted using a maximum of eight extraction cycles of 72 h at a temperature of 37 °C until no more TPO eluates were detected by high-performance liquid chromatography (HPLC). The margin of safety (MoS) was calculated as the ratio between the Derived No-Effect Level (DNEL) and the estimated exposure based on the amount of TPO extracted. **Results**: The total amount of extracted TPO was the lowest for LuxaPrint Ortho Plus (Mean ± SD; 44.0 ± 17.1 ng/mL), followed by optiprint splint (80.6 ± 21.1 ng/mL), FREEPRINT splint 2.0 (127.4 ± 25.3 ng/mL), FREEPRINT ortho (2813.2 ± 348.0 ng/mL), V-Print splint comfort (33,424.6 ± 8357.9 ng/mL) and KeySplint Soft (42,083.5 ± 3175.2 ng/mL). For all tested materials, the calculated MoS was above the critical value of 1, demonstrating toxicological safety in the cured, clinically relevant state. **Conclusions**: Large differences in the residual TPO content were observed between the materials. Although the TPO content in the uncured state may exceed toxicological safety limits, appropriate curing of the investigated materials resulted in a significant reduction in TPO elution and, thus, in products with a very low toxicological risk for the patient.

## 1. Introduction

The incorporation of additive manufacturing into dentistry has developed rapidly in the past years and is becoming more widely accepted and used in dental practices [1,2,3]. Additive manufacturing of dental and orthodontic appliances from 3D models, such as occlusal splints or surgical guides, allows for a wider selection of materials and the fabrication of more sophisticated and complex structures compared to conventional and subtractive manufacturing [4,5]. Integration of 3D printing also enables fully digital workflows, including digital 3D modeling with precise and rapid translation of the treatment plan, improved patient comfort and a reduction in storage space and waste [6,7,8]. However, the clinical outcome of 3D printed devices depends on a variety of factors, such as the type of printer and processing parameters, the design of the dental device or the light-curing resin material used for the manufacturing process [4,9].

Occlusal splints are used for a variety of treatments, such as temporomandibular disorders or for bruxism arising from excessive occlusal loads due to tooth grinding [10,11]. To achieve this, it is important that the splints produced have an exact fit and can withstand the occlusal load during clinical use without failure. Initial studies have shown that 3D-printed dental appliances result in clinically sufficient accuracies when compared to CAD/CAM-milled or thermoformed devices [12,13]. However, the biocompatibility and elution behavior of additively manufactured dental and orthodontic appliances might be critical since the elution of cytotoxic substances is considered higher compared to other manufacturing techniques [14]. The toxicological risk of additively manufactured dental appliances due to uncured residual monomers is influenced by several factors such as type of methacrylate monomers, light curing parameters, temperature, type and concentration of the photoinitiator system [15,16]. Residual monomers and additives can leach out of the cured materials and may harm the surrounding tissues when placed intraorally [17]. Therefore, it is important to investigate the elution behavior of additively manufactured occlusal splint materials under simulated oral conditions [18].

Within the photopolymerization reaction, photoinitiators play a key role in initiating the radical-based reaction that has a considerable impact on the final characteristics of the resulting medical device. These properties encompass the degree of conversion and the mechanical attributes of the final product [19,20]. Diphenyl (2,4,6-trimethylbenzoyl) phosphine oxide (TPO) is an organophosphorus substance that is frequently used as a Norrish type I photoinitiator in resin-based dental materials [21,22,23]. The benefits of TPO are a relatively low impact on color alterations of the final product and a better solubility in most monomers than other Norrish type 1 initiator systems like phenylbis (2,4,6-trimethylbenzoyl) phosphine oxide (BAPO) [24]. However, TPO also exhibits a few drawbacks, such as a lower degree of conversion than BAPO and a higher cytotoxicity compared to camphorquinone (CQ), which can be critical after insufficient light curing given the risk of uncured monomers and additives being released [25].

Dental appliances manufactured by 3D printing are only inserted in a fully polymerized state, thus minimizing the possibility of residual substances that potentially harm the patient. As the manufacturer’s specifications only provide data on the TPO content of the liquid resin used for the additive manufacture of occlusal splints, there is a lack of information regarding the clinically more relevant residual TPO content of the cured material. TPO was recently reclassified by the European Union’s REACH regulation (Registration Evaluation Authorization of Chemicals) with subsequent labeling as a CMR (Carcinogenic, Mutagenic, Reprotoxic) substance of “very high concern”. Consequently, further use of TPO in concentrations exceeding 0.1 wt.% must be justified by the individual manufacturer according to the European Medical Device Regulation (MDR). Accordingly, the objective of this study was to analyze the residual TPO content of a range of 3D-printed light-cured resins used to fabricate occlusal dental splints following extraction under simulated oral conditions. We aimed to ascertain the estimated toxicological risk based on the quantity of TPO extracted to guarantee the patient safety of the medical devices in question.

## 2. Materials and Methods

Six resin-based 3D-printing materials intended to fabricate dental occlusal splints containing TPO were analyzed: LuxaPrint Ortho Plus (DMG, Hamburg, Germany), FREEPRINT splint 2.0 (DETAX, Ettlingen, Germany), optiprint splint (dentona, Dortmund, Germany), KeySplint Soft (Keystone Industries GmbH, Singen, Germany), FREEPRINT ortho (DETAX), V-Print splint comfort (VOCO, Cuxhaven, Germany). The TPO content of the uncured liquid resin materials was determined in single measurements using high-performance liquid chromatography (HPLC, Thermo UltiMate 3000, Thermo Fisher Scientific, Waltham, MA, USA) with a column temperature of 20 °C (250/4 LiChrospher^®^.RP select B (5 µm), Merck KGaA, Darmstadt, Germany). Gradient elution was conducted using water and methanol as eluents at a flow rate of 1.0 mL/min (Table 1).

**Figure 1:**
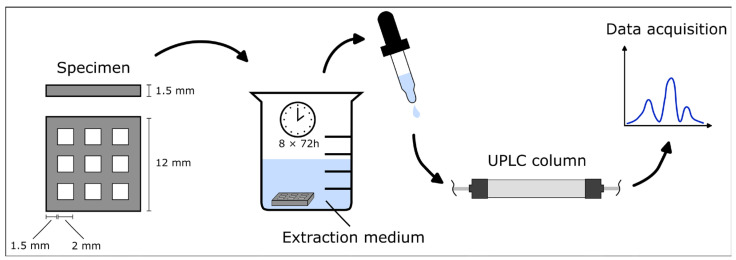
Schematic of the specimen dimensions and the extraction protocol.

Grid-shaped specimens with an edge length of 12 mm and a grid thickness of 1.5 mm were produced using 3D printers in accordance with the recommended workflows of the respective manufacturers for each material (n = 18) (Figure 1).

The specimens were printed using either the ASIGA MAX UV (Asiga, Alexandria, Australia) or the rapidshape D20 II (Rapid Shape GmbH, Heimsheim, Germany) with a working wavelength of 385 nm (Table 2). Subsequent to the printing process, the specimens were subjected to cleaning and post-curing in accordance with the instructions provided by the respective manufacturers. The post-curing procedure was conducted utilizing a xenon flash apparatus (Otoflash G171, NK-Optik, Baierbrunn, Germany) with 2 × 2000 flashes (385 nm) under inert gas conditions.

The extraction protocol was carried out using six parallel extractions for each material, with three samples per extraction solution. According to ISO 10993-17 [26], each specimen, with a surface area of 3.96 cm^2^, was extracted in 1.32 mL of the extraction medium to obtain the specified surface-volume ratio of 3 cm^2^: 1 mL. The cured specimens were extracted in cycles of 72 h at 37 °C, and the TPO content was measured after each cycle using ultra-performance liquid chromatography (UPLC MS-Tof: Xevo G2-Xs QTof, Waters GmbH, Eschborn, Germany). A column temperature of 50 °C was employed, along with gradient elution utilizing a combination of water and acetonitrile, with a flow rate of 0.5 mL/min (Table 1). These conditions were used for analysis using an Acquity BEH C8 1.7 µm column (Waters GmbH, Eschborn, Germany). TPO was identified and quantified through a comparison with a reference compound of known concentration, which was measured under the same analytical conditions. The extraction was repeated eight times or until no further TPO eluates could be detected. Extraction was performed in a solution consisting of water (CAS 7732-18-5; Biosolve Chimie, Dieuze, France) and isopropyl alcohol (CAS 67-63-0; Biosolve Chimie) with a volume ratio of 144.4:154.1 mL and a resulting polarity index of 8.94, acting as saliva simulant. To determine the daily exposition, the 24 h concentration of TPO was calculated from the highest single value measured for each material during one 72 h extraction cycle.

The Margin of Safety (MoS) was calculated as the ratio between the Derived No-Effect Level (DNEL) and the estimated exposure based on the amount of TPO extracted during 24 h. To calculate the daily exposition to a child with a body weight (bw) of 10 kg, a surface area of the manufactured occlusal splint of 90 cm^2^ was assumed. A DNEL of 83.3 µg/kg body weight/day was used according to the “General Population—Hazard via oral route” for TPO (CAS 75980-60-8) provided by the European Chemicals Agency. The derived value in which no effects are expected with a daily intake of 83.3 µg/kg bw/day for oral intake is based on a sub-chronic oral toxicity study in rats in which a NOAEL (no-observed-adverse-effect level) of 50 mg/kg bw/day was determined. A DNEL was then derived with an overall assessment factor of 600 [27]. According to ISO 10993-18 [28], the use of an uncertainty factor (UF) of 2 for chromatographic determinations and a UF of 1.5 for the extraction protocol is required for the chemical characterization of analytical data. The calculated MoS was, therefore, divided by 3.

Statistical analysis was performed using GraphPad PRISM (Version 9, GraphPad Software Inc., San Diego, CA, USA). The total extracted TPO content and the maximum TPO content of one single extraction cycle were compared using the non-parametric Kruskal–Wallis test and pairwise comparisons (Dunn’s test). For this, the values of the 3 samples within each parallel extraction approach were averaged. A Type I error level of 0.05 was used for all tests of significance.

## 3. Results

The TPO content of the resin splint materials under investigation was found to be between 1% and 1.5% in the liquid state (Table 3), which is in accordance with the specifications set out by the manufacturers. This ensured a comparable baseline for the subsequent extraction protocol.

**Figure 2:**
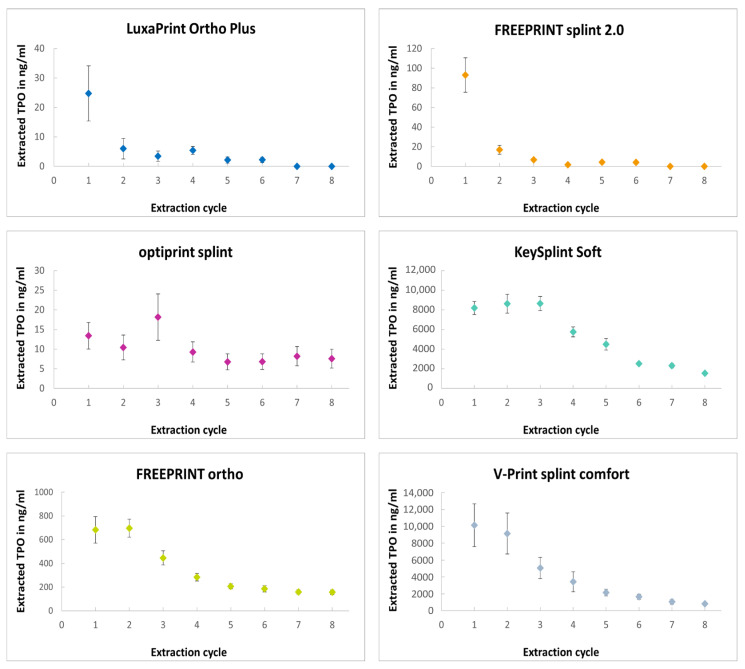
Progression of the TPO extracted from the cured resin materials as a function of the extraction cycle.

Ranking of the materials according to the total amount of TPO extracted from the cured specimens was as follows (low to high): LuxaPrint Ortho Plus—optiprint splint—FREEPRINT splint 2.0—FREEPRINT ortho—V-Print splint comfort—KeySplint Soft. It was observed that the amount of extracted TPO decreased for each consecutive extraction cycle (Figure 2). The maximum TPO content extracted was determined for LuxaPrint Ortho Plus, FREEPRINT splint 2.0 and V-print splint comfort following the initial extraction cycle. For the remaining materials, a progressive increase in TPO content was observed with subsequent extraction cycles, followed by a subsequent decline. For LuxaPrint Ortho Plus and FREEPRINT splint 2.0, no more TPO could be detected after the sixth extraction cycle. For all tested materials, the calculated MoS for a child with a body weight of 10 kg was over the critical value of 1, demonstrating toxicological safety in the cured, clinically relevant state.

## 4. Discussion

Polymerization is a process whereby liquid resin monomers or oligomers react with one another to form solid polymers. The polymerization reaction in 3D printing is initiated by light, using specialized photoinitiators such as TPO, which generate free radicals upon irradiation with a suitable wavelength. The extent to which the monomers react in the radical polymerization to form the cured material is called the degree of conversion [29]. The degree of conversion is of paramount importance in the context of light-curing resin materials, as it has the potential to influence a number of crucial characteristics, including the mechanical and physical properties of the medical device in question [30,31]. Norrish type I photoinitiators, such as TPO or BAPO, are typically characterized by higher curing efficiency compared to Norrish type II photoinitiators, such as CQ. This is particularly evident when working with low concentrations, and they are therefore commonly employed in 3D printing dental materials [29,32,33,34]. The use of TPO as a photoinitiator in 3D-printed dental appliances is further supported by the favorable color stability and surface hardness of the cured material in comparison to resins containing BAPO and CQ [35,36,37]. Apart from the negative impact on the mechanical properties, incomplete conversion due to improper photopolymerization can result in the release of residual additives, monomers, and oligomers from additively manufactured dental appliances, which may potentially affect cell viability. In particular, TPO was found to have a dose-dependent cytotoxic effect on human oral keratinocytes and V79 cells, with a higher level of toxicity compared to CQ [37]. As the photoinitiator system was identified as one of the main components in extracts of resin-based materials [17], this study aimed to analyze the residual TPO content in resin-based materials used to additively fabricate occlusal splints.

In order to ascertain the residual TPO content of the splint materials, a solution of water and isopropanol with a polarity of 8.94 was employed as the extraction medium, as opposed to the use of artificial saliva or other electrolyte solutions. The polarity index is a measure of the dissolution behavior of substances, both polar and non-polar. As such, it is assumed to be a useful indicator when selecting an appropriate extraction medium to simulate the conditions of the oral cavity. As saliva normally consists of 98% water with a polarity of 10.2 according to ISO 10993-18, isopropanol with a polarity of 3.9 was added to the extraction tests to simulate a worst-case scenario of real oral conditions. The resulting polarity of the extraction medium was approximately one scale point below the assumed polarity of saliva. Thus, the measured residual levels of TPO that could be extracted are theoretically higher than those that are expected by contact with saliva.

Large differences in the extracted TPO content were observed between the dental splint materials investigated in this study. Whereas LuxaPrint Ortho Plus and optiprint splint showed a mean total amount of extracted TPO of <100 ng/mL after eight extraction cycles, KeySplint Soft and V-Print splint comfort resulted in a mean extracted TPO content of >30,000 ng/mL. Given that the baseline TPO content of the liquid resins was similar between the materials, this discrepancy may be attributed to the different formulations of the resins. Previous research has demonstrated that the selection of resin monomers can influence the solubility of the photoinitiator and the degree of conversion during photopolymerization [38,39]. However, as no information on the detailed composition of the materials used was available, no reliable conclusions on the specific factors affecting the residual TPO content after curing could be drawn. Besides the material composition, a processing workflow that is optimized for the corresponding material is crucial for the additive fabrication of a dental appliance that is safe for use in a toxicological context. Wulff et al. highlighted in their study that post-processing parameters such as the cleaning protocol and post-curing procedure can significantly influence the cytotoxicity of 3D-printed dental splints [40]. In their in vitro study with RAW264.7 mouse macrophages that were exposed to extracts of the printed disc-shaped specimens, it could be demonstrated that LuxaPrint Ortho Plus showed a significantly higher cell survival rate compared to another material used to fabricate occlusal splints. These findings are in line with the results of the present study, which shows that LuxaPrint Ortho Plus was among the materials with the lowest residual TPO content that could be extracted from the cured specimens. Despite the TPO content in the uncured liquid state of the investigated resin materials exceeding the regulatory limits (>0.1 wt.%) as defined by the MDR for CMR substances, the results of this study demonstrate that appropriate curing and post-processing of the used materials resulted in a significant reduction in the residual TPO content that could be extracted. It can thus be concluded that the medical devices derived from the aforementioned resin materials present a negligible toxicological risk for the patient concerning TPO when the recommended workflow parameters set forth by the manufacturer are duly adhered to.

An ideal photoinitiator of 3D printing resin should have biocompatibility, color stability, a high degree of conversion and 3D printing accuracy. However, these properties vary depending on the photoinitiator system used, thus affecting the additively fabricated dental appliance. In a recent study, Kim and colleagues demonstrated that TPO-L can effectively address the challenges associated with the cytotoxicity of TPO and BAPO when employed as a photoinitiator for the 3D printing of dental resins [19,41]. Other promising novel photoinitiators include germanium compounds and acylphosphine oxides [16,29,42]. The exchange of initiators for reasons related to toxicological concerns is possible in specific circumstances, but this may result in limitations in processing or discrepancies regarding the product properties. During the material development phase, a number of crucial factors must be taken into account when selecting an appropriate photoinitiator. These include the type of monomers, color stability, solubility of the photoinitiator in the mixture and the required curing speed. The advancement of LED technology has also had a notable impact on the development of photopolymerized materials [20].

The objective of this study was to investigate the potential toxicological risks associated with polymerized occlusal splints for the patient. However, the environmental impact of printable resins in dentistry, which involves energy consumption, waste materials and environmental pollution, is also of paramount importance [43,44]. In particular, with regard to the additive manufacturing of orthodontic appliances, the issue of waste material represents a significant challenge that must be addressed, given the considerable negative environmental impact of the excess waste material produced by current techniques. To date, there is a paucity of documentation concerning the environmental impact of printable resins utilized in the production of 3D-printed dental appliances. In order to ensure the safe manufacture and disposal of these materials by dental personnel, a statement is included in the Instructions For Use of the respective manufacturer indicating that the resin mixture should be incinerated as hazardous waste in accordance with official regulations. Additionally, protective clothing is required when handling these resin materials.

A limitation of this study is that the influence of the extracted TPO on cell viability was not investigated. Consequently, the results of this study should be interpreted with caution, as the calculated MoS is based on theoretical considerations. It is also important to highlight that the inappropriate fabrication of 3D-printing resins with parameters that are not aligned with the recommendations provided by the manufacturers may lead to an elevated risk of uncured monomers and additives that can potentially leak from the materials, thereby increasing the likelihood of complications associated with dental appliances. Subsequent studies should also investigate the use of 3D-printing dental resins with alternative photoinitiator systems, with a view to comparing the potential toxicological risks associated with the materials. Furthermore, a detailed chemical characterization of the materials under investigation is required to facilitate more reliable statements on the potential factors leading to significant variations in residual TPO content. The issue of missing compositions could be addressed in subsequent parameter studies through the utilization of a standardized reference material. As the post-processing cleaning procedure of photopolymerized dental appliances has the potential to influence the cytotoxicity of the manufactured device [40], soaking the occlusal splints could be an effective method for extracting residual TPO prior to patient use. This should be a key consideration in subsequent studies.

## 5. Conclusions

Large differences in the residual TPO content were observed between the resin-based materials intended to additively fabricate occlusal splints. Although the TPO content in the uncured liquid state may exceed regulatory limits, appropriate curing of the investigated materials according to the instructions provided by the manufacturer resulted in a significant reduction in the extracted TPO and, thus, in medical devices with a very low toxicological risk for the patient. It is, therefore, essential to adhere to the manufacturer’s specifications when curing 3D-printed materials to ensure a sufficient conversion rate and minimize the potential risk of exposure to uncured monomers or additives that may cause harm to the patient.

## Figures and Tables

**Table 1:** HPLC and UPLC gradient elution parameters to analyze the TPO content of the uncured liquid resins and the residual TPO content of the cured specimens after extraction.

HPLC—Uncured Resins			UPLC—Cured Specimens		
Time [min]	Methanol [%]	Water [%]	Time [min]	Acetonitrile [%]	Water [%]
0	70	30	0	30	70
0.5	70	30	0.5	30	70
14.5	95	5	6	50	50
15	70	30	7	50	50
20	70	30	16	90	10
			17	30	70
			20	30	70

**Table 2:** TPO content of the used resin materials stated in the Material Safety Data Sheet (MSDS) provided by the manufacturer and fabrication parameters according to the Instructions For Use (IFU).

	TPO (MSDS)	Printer	Cleaning (IFU)	Post-Curing (IFU)
LuxaPrint Ortho Plus (DMG)	1–2%	Asiga MAX UV	2 + 2 min with isopropanol in an ultrasonic bath	2 × 2000 flashes
FREEPRINT splint 2.0 (DETAX)	1–5%	Asiga MAX UV	2 + 3 min with isopropanol in an ultrasonic bath	2 × 2000 flashes
optiprint splint (dentona)	<2.5%	Asiga MAX UV	2 + 3 min with isopropanol in an ultrasonic bath	2 × 2000 flashes
KeySplint Soft (Keystone)	<3%	rapidshape D20 II	2 + 3 min with isopropanol in an ultrasonic bath	2 × 2000 flashes
FREEPRINT ortho (DETAX)	1–2.5%	Asiga MAX UV	5 + 3 min with isopropanol in an ultrasonic bath	2 × 2000 flashes
V-Print splint comfort (VOCO)	1–5%	Asiga MAX UV	2 + 3 min with isopropanol in an ultrasonic bath	2 × 2000 flashes

**Table 3:** TPO content of the resin materials in the liquid state and after photopolymerization and extraction. Superscript letters indicate statistically significant differences between the materials.

	LuxaPrint Ortho Plus (DMG)	FREEPRINT Splint 2.0 (DETAX)	Optiprint Splint (Dentona)	KeySplint Soft (Keystone)	FREEPRINT Ortho (DETAX)	V-Print Splint Comfort (VOCO)
Liquid resin TPO [%]	1.04	1.47	1.18	1.19	1.19	1.00
Total TPO, 8 × 72 h [ng/mL]	44.0 ± 17.1 ^a^	127.4 ± 25.3 ^ab^	80.6 ± 21.1 ^ab^	42,083.5 ± 3175.2 ^c^	2813.2 ± 348.0 ^bc^	33,424.6 ± 8357.9 ^c^
Maximum TPO, 72 h [ng/mL]	24.8 ± 9.4 ^a^	93.2 ± 17.5 ^ab^	18.1 ± 5.9 ^ab^	8640.4 ± 714.1 ^c^	696.0 ± 76.3 ^bc^	10,135.2 ± 2542.2 ^c^
Maximum TPO, 24 h [ng/mL]	8.3 ± 3.1	31.1 ± 5.8	6.0 ± 2.0	2880.1 ± 283.0	232.0 ± 25.4	3378.4 ± 847.4
Daily exposition, 10 kg bw [µg/kg bw/d]	0.025 ± 0.009	0.093 ± 0.017	0.018 ± 0.006	8.640 ± 0.849	0.696 ± 0.076	10.135 ± 2.542
Margin of Safety (MoS)	1115 > 1	299 > 1	1543 > 1	3 > 1	40 > 1	3 > 1

## Data Availability

The data that support the findings of this study are available from the corresponding author upon reasonable request.
